# Susceptibility to cyflumetofen in populations of the citrus leprosis mite, *Brevipalpus yothersi* , from Brazilian citrus orchards

**DOI:** 10.1002/ps.70262

**Published:** 2025-09-28

**Authors:** Hector Alonso Escobar‐Garcia, Daniel Júnior de Andrade

**Affiliations:** ^1^ São Paulo State University (UNESP), School of Agricultural and Veterinary Sciences Jaboticabal Brazil; ^2^ Universidad Nacional de Piura (UNP), Facultad de Agronomía Piura Peru

**Keywords:** ArcGIS, chemical control, diagnostic concentration, flat mites, oviposition reduction

## Abstract

**BACKGROUND:**

*Brevipalpus yothersi* Baker (Acari: Tenuipalpidae), the citrus leprosis mite, is currently the most economically significant mite pest in commercial citrus orchards, serving as the vector of citrus leprosis virus C (CiLV‐C, *Cilevirus leprosis*). Its control has traditionally relied on the intensive use of synthetic acaricides, raising concerns regarding field efficacy and resistance development. To support integrated pest management (IPM) and resistance management strategies, monitoring the susceptibility of field populations is essential. Cyflumetofen, an acaricide that inhibits mitochondrial complex II electron transport, offers a distinct mode‐of‐action (MoA) for mite control. This study aimed to characterize the susceptibility of *B. yothersi* populations to cyflumetofen to evaluate its potential use in sustainable resistance management and prolong its efficacy in commercial applications.

**RESULTS:**

Field populations exhibited high susceptibility to cyflumetofen, with median lethal concentration (LC_50_LC₅₀) values ranging from 0.36 to 1.31 mg L^−1^ and 95% lethality (LC_95_) values from 1.22 to 4.99 mg L^−1^. Resistance levels were low, with resistance ratios below 2.79‐fold at LC_50_ and below 3.06‐fold at LC_95_, relative to a susceptible reference population.

**CONCLUSION:**

Cyflumetofen proved effective against *B. yothersi* in the surveyed regions, with field populations showing high susceptibility. To maintain its efficacy and extend its commercial longevity, rotation with other synthetic acaricides featuring different MoAs is recommended as part of a resistance management plan. © 2025 The Author(s). *Pest Management Science* published by John Wiley & Sons Ltd on behalf of Society of Chemical Industry.

## Graphical abstract

*Brevipalpus yothersi*, the vector of citrus leprosis virus, showed high susceptibility to cyflumetofen. Its low resistance suggests it is effective, but should be rotated with other acaricides with different modes‐of‐action for sustainable management.

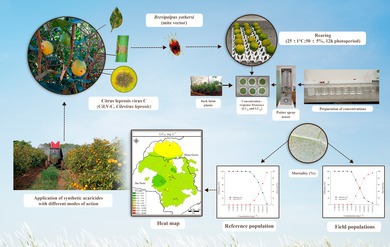

## 1 INTRODUCTION

*Brevipalpus yothersi* Baker (Acari: Tenuipalpidae), a polyphagous flat mite, infests >112 plant species across 52 botanical families. It is one of the primary mite pests affecting sweet orange crops in southeastern Brazil and throughout much of the Americas.^1^ Despite advances in integrated pest management (IPM), chemical control via foliar spraying of synthetic acaricides remains the most widely adopted and effective strategy for suppressing *B. yothersi* populations during critical fruiting stages.^2^, ^3^ Brazil currently holds one of the largest acaricide markets globally, with usage increasing steadily in recent years.^4^, ^5^, ^6^ However, frequent and repeated applications of acaricides with identical modes‐of‐action (MoAs) have led to resistance development in *B. yothersi* populations, threatening long‐term control efficacy. This risk is exacerbated by the species' biological traits, including high fecundity, thelytokous parthenogenetic reproduction, and a rapid life cycle, enabling ≤14 generations per year.^7^, ^8^

Currently, 34 synthetic acaricides are registered by ProteCitrus for *B. yothersi* control in Brazil, encompassing various chemical classes and MoAs.^6^, ^9^ Nonetheless, resistance has been reported in field populations to several active ingredients, including dicofol, hexythiazox, propargite, lime sulfur, sulfur and spirodiclofen.^8^, ^10^, ^11^, ^12^, ^13^, ^14^, ^15^, ^16^, ^17^, ^18^ In this scenario, cyflumetofen has emerged as a promising alternative. This contact acaricide belongs to the benzoyl acetonitrile group and functions as a mitochondrial complex II electron transport inhibitor (IRAC subgroup 25A). Since its global market introduction in 2007 and its registration in Brazil in 2014, cyflumetofen has demonstrated strong efficacy against multiple mite species.^4^, ^5^, ^9^, ^19^, ^20^, ^21^

Despite its registration, no prior studies in Brazil have evaluated the susceptibility of *B. yothersi* populations to cyflumetofen. The populations assessed in this study were previously established and maintained under controlled conditions at the Acarology Laboratory of UNESP (Jaboticabal, Brazil), having also been used in earlier research.^16^ Here, we present toxicity bioassays conducted on adult females of *B. yothersi*. The main aim of this study was to assess the susceptibility of geographically distinct *B. yothersi* populations to cyflumetofen. The specific objectives were: (i) to generate baseline data on resistance levels to cyflumetofen, and (ii) to determine a diagnostic concentration for potential field monitoring. The outcomes of this study are intended to contribute to improved IPM and resistance management (RM) strategies in Brazilian citrus production.

## 2 MATERIALS AND METHODS

### 2.1 Acquisition and maintenance of *B. yothersi* populations

Adult females of *Brevipalpus yothersi* used in the bioassays originated from populations maintained on green fruits of sweet orange cultivars ‘Valencia’ or ‘Navel’, both grafted onto Swingle citrumelo rootstock, as described by Escobar‐García and Andrade.^16^ These populations were reared in a climate‐controlled room at 25 ± 1 °C, 50 ± 5% relative humidity and a 12 h:12 h, light:dark photoperiod. One population, designated as the susceptible reference (Laboratory‐AcaroLab, UNESP, Jaboticabal, Brazil), has been continuously maintained on sweet orange fruit since 2010 without exposure to synthetic acaricides.

**Figure 1:**
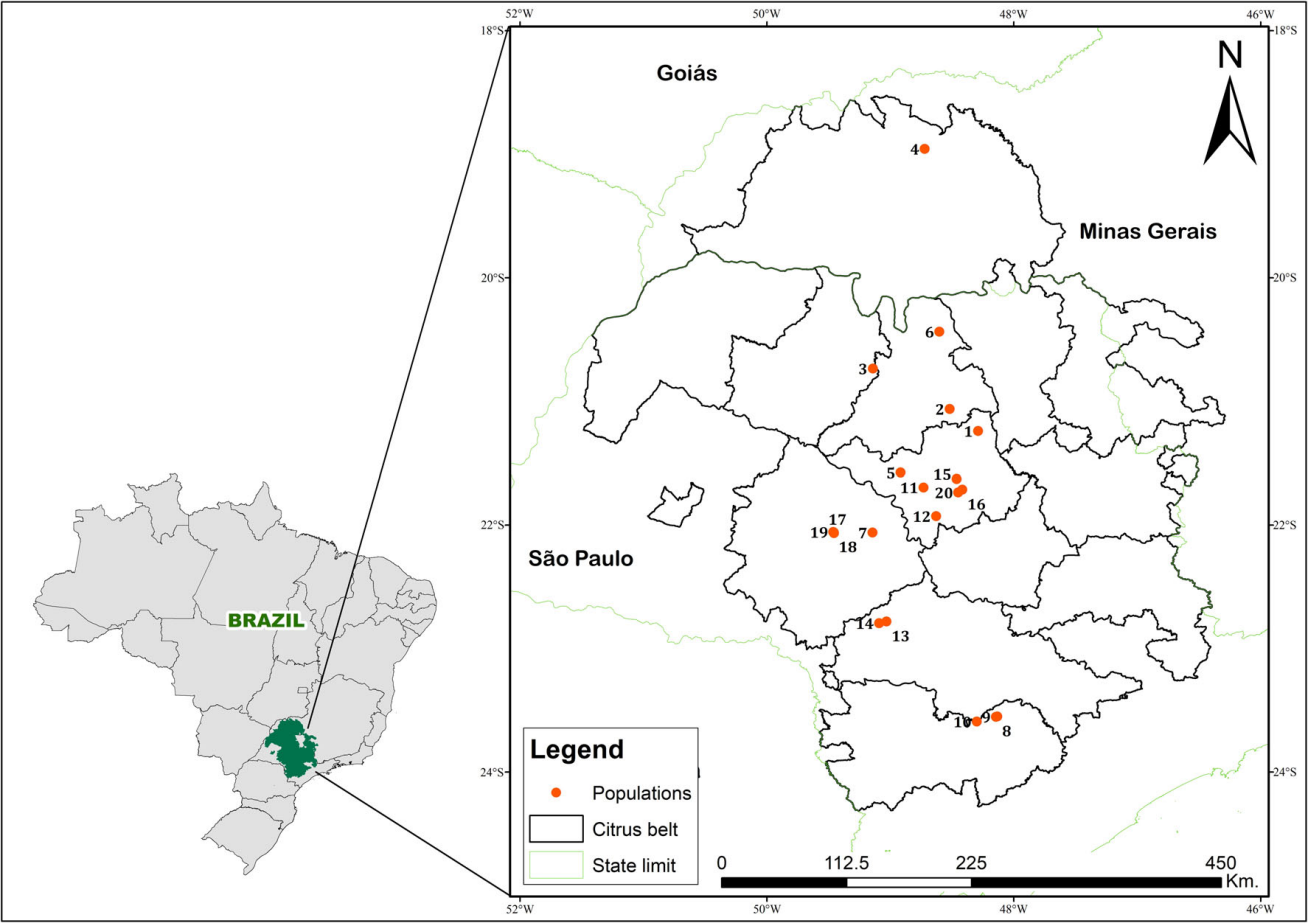
Sites of *Brevipalpus yothersi* population sampling. Sampling site numbers correspond to those listed in Table 1.

The remaining 19 populations were collected from commercial citrus orchards between February 2023 and April 2024, across 14 citrus‐producing municipalities representing major regions of the Brazilian citrus belt (Fig. 1; Table 1). Although detailed historical spray records were not available for each orchard, information obtained from growers and regional technical reports indicates that propargite, hexythiazox, spirodiclofen and spiromesifen are the most frequently applied acaricides in these production areas. Cyflumetofen was registered in 2014 for citrus in Brazil, and its use in the sampled orchards before collection was minimal or absent. All females were morphologically identified as *B. yothersi* based on the criteria established by Beard *et al*.^22^ Permanent microscope slides containing voucher specimens from each population were deposited in the Acari Collection (DZB) at the Department of Zoology and Botany, UNESP, São José do Rio Preto, São Paulo, Brazil.

**Table 1:** Background information on the *Brevipalpus yothersi* field populations used in this study

No	Population	Latitude and longitude	Rootstock	Variety	Mite detection year
1	Laboratory‐AcaroLab	21°14′25.06” S 48°17′19.42” W	Various	Various	2010
2	Bebedouro	21°3′45.20” S 48°31′7.50” W	Rangpur Lemon	Valencia	2009
3	Guapiaçu	20°44′6.65” S 49°8′28.57” W	Swingle Citrumelo	Pera	2015
4	Monte Alegre de Minas	18°57′20.21” S 48°43′20.00” W	Swingle Citrumelo	Valencia	2012
5	Itápolis	21°34′31” S 48°55′00” W	Swingle Citrumelo	Natal	2005
6	Barretos	20°26′13” S 48°36′08” W	Swingle Citrumelo	Valencia	2018
7	Reginópolis	22°03′34.7” S 49°08′36.6” W	Sunki Tangerine	Pera	2013
8	Itapetininga‐204	23°33′00.4” S 48°08′03.5” W	Swingle Citrumelo	Hamlin	2017
9	Itapetininga‐206	23°33′12.2” S 48°08′40.2” W	Swingle Citrumelo	Hamlin	2017
10	Itapetininga‐305	23°35′26.2” S 48°17′57.5” W	Swingle Citrumelo	Valencia	2008
11	Tabatinga	21°41′43” S 48°43′51” W	Tangerine Cleópatra	Hamlin	2005
12	Boa Esperança do Sul	21°55′38.4” S 48°37′46.4” W	Sunki Tangerine	Pera	2018
13	Iaras‐191	22°46′50.2” S 49°01′53.0” W	Rangpur Lemon	Valencia	2007
14	Iaras‐253	22°47′39.1” S 49°05′26.5” W	Lime Volkamerian	Valencia	2007
15	Matão	21°37′41.5” S 48°27′46.5” W	Swingle Citrumelo	Valencia	2004
16	Gavião Peixoto	21°42′44.38” S 48°25′2.93” W	Lime Volkamerian	Valencia	2006
17	Pirajuí‐501	22°3′53.85” S 49°27′19.14” W	Swingle Citrumelo	Pera	2017
18	Pirajuí‐502	22°3′31.53” S 49°27′29.69” W	Swingle Citrumelo	Pera	2017
19	Pirajuí‐509	22°3′21.89” S 49°27′44.31” W	Swingle Citrumelo	Valencia	2017
20	Nova Europa	21°44′07.1” S 48°27′00.3” W	Swingle Citrumelo	Valencia	2021

Each population was reared on six to eight green citrus fruits placed on plastic trays designed to hold chicken eggs, which served as supports. Each tray‐fruit set constituted an experimental unit and was labeled according to its geographic origin (Table 1). Populations were maintained for 1–2 months, which allowed for the collection of 1650 adult females per population for use in the bioassays described in the following section.

### 2.2 Concentration–response bioassays

In this study, the synthetic acaricide Obny® 200 SC, a commercial suspension concentrate (SC) formulation containing 200 g L^−1^ cyflumetofen, was used. The product was manufactured by UPL (Ituverava, Brazil). The response of *B. yothersi* populations to cyflumetofen was evaluated through direct‐contact bioassays on adult females.

Bioassays were performed using leaves of jack bean, *Canavalia ensiformis* (L.) DC. (Fabaceae), cut into 80‐mm‐diameter discs. Jack bean plants were cultivated in a glasshouse in pots (255 × 200 × 144 mm) filled with a substrate mixture of clay soil, cattle manure and sandy soil in a 1:1.5:1 ratio. Plants were grown for 1 month without pesticide exposure and irrigated twice weekly. Leaf discs were placed abaxial side up in glass Petri dishes (90 mm diameter × 14 mm height) over a 10‐mm‐thick moistened foam layer (with deionized water), topped with a 1‐mm layer of cotton. The edges of the discs were surrounded by moist cotton to maintain leaf turgor and prevent mite escape. Each dish was labeled according to concentration and replicate.

Ten concentrations of cyflumetofen were prepared from the recommended field rate (80 mg L^−1^) by serial dilution: 80, 40, 20, 10, 5, 2.5, 1.25, 0.62, 0.31 and 0.15 mg L^−1^. Deionized water was used as the solvent. Each bioassay (temporal replicate) comprised 11 treatments (10 concentrations plus a deionized‐water control). For each treatment within a temporal replicate, we used three independent arenas (subsamples) consisting of leaf‐disc dishes with 25 adult females each (*n* = 75 per treatment per bioassay date). The entire bioassay was replicated in time on a separate date under identical environmental and procedural conditions (two temporal replicates in total). Consequently, for each population and each treatment, the overall sample size was six arenas (150 females), totaling 1650 females per population (11 treatments × 150 females). Mites were transferred onto the leaf discs using a fine‐tipped brush.

Treatments were applied 30 min after mite transfer using a Potter spray tower (Burkard Manufacturing Co. Ltd, Rickmansworth, UK). Each Petri dish received 2 mL of solution at 68.95 kPa, ensuring uniform coverage and an average deposition of 1.56 mg cm^−2^. Dishes were then placed in plastic trays (435 × 296 × 75 mm) and maintained under the same environmental conditions used for rearing. Treatments were applied from the lowest to the highest concentration. The spray tower was rinsed with deionized water and dried with a paper towel between treatments to prevent cross‐contamination.

Mortality was assessed 48 h post‐treatment by counting live and dead females. At the end of the exposure period, the 25 females from each Petri dish were gently grouped at the center of the leaf disc using a fine brush. Females were considered alive if they exhibited vigorous, coordinated leg movements; those immobile after gentle stimulation were considered dead. Oviposition also was recorded and expressed as a percentage relative to that of the control group for each population. The 48‐h assessment interval was selected based on the known persistence of cyflumetofen biological activity against *B. yothersi* for ≤12 days postexposure (dpe), and its appropriateness for Probit analysis. For the extended 12‐day assessment of cyflumetofen residual biological activity, only the laboratory reference population was used. After spraying females with each tested concentration in the Potter spray tower, mortality was recorded at 1, 2, 4, 7 and 12 dpe. All evaluations were conducted at 14:00 h, matching the initial spraying time, to ensure consistency and minimize potential circadian effects.

### 2.3 Diagnostic concentration for monitoring the susceptibility of field populations of *B. yothersi*

Susceptibility monitoring of the 19 field populations of *B. yothersi* to cyflumetofen was conducted in comparison with the reference susceptible population (Laboratory‐AcaroLab; Population 1 in Table 1). The diagnostic concentration was established based on the LC_95_ value estimated for the reference population, which was 1.63 mg L^−1^. Females from each population were exposed to this concentration to estimate survival percentages.

The experiment followed a completely randomized design with six arenas per population. Each arena consisted of 25 females, totaling 150 females evaluated per population. Survival was assessed 48 h postexposure to the diagnostic concentration. During the same 48 h period, oviposition was recorded for each population. Relative oviposition was then calculated as the percentage of mean eggs per female in the treatment compared to the mean eggs per female in the deionized‐water control, using the formula:

$$\text{Relative oviposition}\;\left(\%\right)=\frac{\text{Mean eggs}/\text{female in treatment}}{\text{Mean eggs}/\text{female in control}\;}\times 100$$

This approach provides a standardized measure of oviposition reduction relative to untreated females.

At the end of the exposure period, females were gently gathered at the center of each leaf disc using a fine‐tipped brush. Individuals were considered alive if they exhibited vigorous, coordinated leg movements; immobile mites, even after gentle stimulation, were considered dead.

### 2.4 Statistical analysis

Probit analysis^23^ was used to evaluate *B. yothersi* mortality data after correcting for control mortality using Abbott's formula.^24^ The analysis was performed using the PROC PROBIT procedure in SAS software.^25^ Variability within each population was assessed using the slope generated from the Probit analysis. Populations with slope values >2 were considered relatively homogeneous, while those with slopes <1 were classified as relatively heterogeneous.^26^

The 95% lethal concentration (LC_95_) estimated for the reference population (Laboratory‐AcaroLab) was used as the diagnostic concentration to monitor susceptibility in the 19 field populations. This choice was made because LC_95_ provides a robust threshold to detect early shifts in susceptibility while minimizing the risk of overestimating resistance in field populations. Higher thresholds such as LC_99_ or the field‐recommended concentration could mask low but emerging resistance by causing near‐complete mortality in all populations, thus reducing sensitivity for early detection. Survival percentage data at this concentration were analyzed using multiple sample comparison tests. Both the parametric Tukey's honestly significant difference (HSD) (multiple range test) and the nonparametric Kruskal–Wallis test were applied to identify significant differences in survival and oviposition means and medians among populations. All statistical analyses were performed using centurion XIX software (StatGraphics Inc., The Plains, VA, USA).^27^ A 5% significance level was adopted for all tests.

Resistance ratios (RR_50_) were calculated by dividing the LC_50_ values of field populations by the LC_50_ value of the reference population (Laboratory‐AcaroLab). Interpretation of RR_50_ followed the classification scale proposed by Fukami *et al*.^28^ values <10 were considered low resistance; 10–40, moderate; 40–60, high; and>60, very high. The 95% confidence intervals (CI) for RR_50_ values were calculated according to the method of Robertson and Preisler.^29^ If the value 1 fell within the CI, there was no significant difference between the field and reference populations. Conversely, if 1 was outside the interval, a significant difference was inferred, indicating that the field population was either more or less resistant than the reference.

Based on survival data at the diagnostic concentration, as well as LC_50_, LC_95_ and RR_50_ values, heat maps were generated using arcgis software.^30^ For the diagnostic concentration, the color scale was based on female survival percentages: green indicated low survival, yellow moderate survival and red high survival. For LC_50_ and LC_95_, the color scale corresponded to tested concentration ranges (0.15–80 mg L^−1^). Red areas represented populations with LC_50_ or LC_95_ values close to or exceeding the field‐recommended concentration (80 mg L^−1^), suggesting the need for higher doses to achieve effective control. Green and yellow areas indicated susceptible populations, which were likely to be manageable with lower concentrations. For RR_50_ values, the color scale was based on resistance potential: green signified low risk, yellow moderate risk and red high risk of resistance to cyflumetofen.

## 3 RESULTS

### 3.1 Concentration–response bioassays

**Figure 2:**
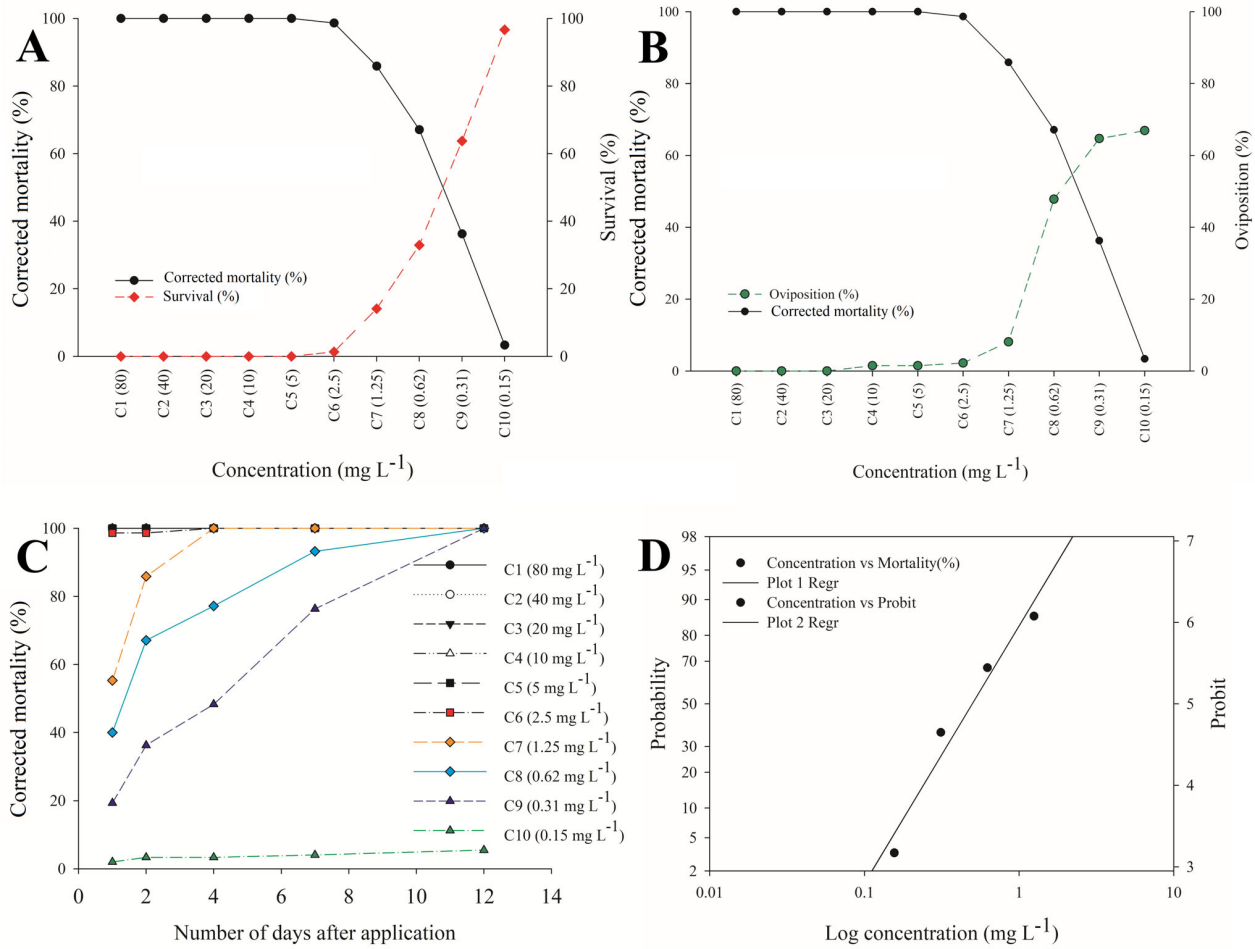
Concentration–response curves of *Brevipalpus yothersi* from the reference population (Laboratory–AcaroLab) reared on *Canavalia ensiformis* and exposed to the acaricide cyflumetofen. (A) Concentration *versus* corrected mortality and concentration *versus* survival curves (red dashed line), evaluated 48 h postexposure. (B) Concentration *versus* corrected mortality and concentration *versus* oviposition curves (green dashed line), evaluated 48 h postexposure. (C) Efficacy of each concentration assessed over a 12‐day period postexposure. (D) Concentration–response curves derived from Probit analysis.

Mortality in the control group for each *B. yothersi* population remained below 5%, allowing for accurate correction of natural mortality in the bioassays. The Probit analysis results are presented in Table 2. The concentration–response data for *B. yothersi* populations exposed to cyflumetofen fitted the Probit model well based on log‐transformed concentrations, as evidenced by low chi‐square (χ^2^) values (1.96 to 12.49) and nonsignificant *P*‐values (0.12 to 0.98). Cyflumetofen exhibited high toxicity against *B. yothersi* females. Detailed data for the reference population (Laboratory‐AcaroLab), exposed to 10 concentrations of the product, are shown in Table 2 and graphically in Fig. 2.

**Table 2:** Probit analysis and median/95% lethal concentration data (95% CI) for *Brevipalpus yothersi* populations after 48‐h exposure to cyflumetofen

No	Population	Slope ±SE*	LC_50_ (mg L^−1^)	95% CI^†^	LC_95_ (mg L^−1^)	95% CI^†^	χ^2^ ^‡^	*P* ^§^	RR_50_ (95% CI)^¶^
1	Laboratory‐AcaroLab	3.06 ± 0.22	0.47	0.42–0.53	1.63	1.37–2.04	7.25	0.51	‐
2	Bebedouro	2.76 ± 0.21	0.36	0.32–0.41	1.43	1.18–1.83	4.03	0.85	0.77 (0.42–1.39)
3	Guapiaçu	2.78 ± 0.19	0.61	0.54–0.68	2.38	1.97–3.00	6.76	0.56	1.30 (0.74–2.29)
4	Monte Alegre de Minas	2.65 ± 0.18	1.19	1.06–1.34	4.99	4.11–6.35	9.77	0.20	2.53 (1.45–4.41)
5	Itápolis	3.36 ± 0.23	0.95	0.86–1.05	2.93	2.49–3.58	11.91	0.16	2.02 (1.09–3.76)
6	Barretos	5.35 ± 0.46	1.31	1.21–1.43	2.67	2.34–3.17	10.24	0.12	2.79 (1.02–7.63)
7	Reginópolis	2.68 ± 0.19	0.41	0.36–0.46	1.69	1.39–2.16	5.76	0.67	0.87 (0.49–1.56)
8	Itapetininga‐204	3.08 ± 0.21	0.65	0.59–0.73	2.24	1.88–2.80	9.21	0.33	1.38 (0.76–2.52)
9	Itapetininga‐206	3.31 ± 0.23	0.55	0.49–0.61	1.73	1.45–2.14	10.00	0.27	1.17 (0.62–2.20)
10	Itapetininga‐305	3.28 ± 0.23	0.62	0.56–0.69	1.97	1.67–2.44	6.76	0.56	1.32 (0.71–2.45)
11	Tabatinga	3.05 ± 0.22	0.45	0.40–0.49	1.55	1.29–1.94	1.96	0.98	0.96 (0.52–1.77)
12	Boa Esperança do Sul	3.52 ± 0.25	0.65	0.58–0.71	1.89	1.61–2.32	7.42	0.49	1.38 (0.72–2.65)
13	Iaras‐191	2.96 ± 0.22	0.82	0.73–0.92	2.95	2.46–3.74	10.25	0.17	1.74 (0.95–3.21)
14	Iaras‐253	2.86 ± 0.19	0.53	0.47–0.59	1.98	1.65–2.49	4.55	0.80	1.13 (0.63–2.02)
15	Matão	3.07 ± 0.25	0.65	0.58–0.73	2.24	1.86–2.84	6.21	0.52	1.38 (0.72–2.66)
16	Gavião Peixoto	2.67 ± 0.18	0.55	0.49–0.62	2.27	1.87–2.90	12.49	0.13	1.17 (0.67–2.05)
17	Pirajuí‐501	3.09 ± 0.23	0.40	0.36–0.45	1.38	1.15–1.74	7.97	0.44	0.85 (0.45–1.60)
18	Pirajuí‐502	3.22 ± 0.24	0.43	0.39–0.48	1.40	1.18–1.75	7.07	0.53	0.91 (0.48–1.73)
19	Pirajuí‐509	3.10 ± 0.24	0.36	0.32–0.40	1.22	1.02–1.54	8.15	0.42	0.77 (0.40–1.46)
20	Nova Europa	3.12 ± 0.23	0.44	0.39–0.49	1.48	1.24–1.86	4.48	0.81	0.94 (0.50–1.75)

Each population was represented by 1650 females in the bioassays.

* Slope and standard error (SE) derived from the Probit analysis.

^†^ Confidence interval of lethal concentration (lower–upper limit) (mg L^−1^).

^‡^ Chi‐square value.

^§^ *P*‐values >0.05 indicate a good fit to the Probit analysis.

^¶^ Resistance ratio (RR_50_) = LC_50_ for the field population / LC_50_ for the reference population (Laboratory‐AcaroLab), including the RR estimate (95% CI) following Robertson and Preisler (1992).

Probit slope values indicated that all populations had a homogeneous structure (slope>2.0). The Barretos population exhibited the highest slope (5.35), whereas the Monte Alegre de Minas population had the lowest (2.65) (Table 2).

The LC_50_ and LC_95_ values for *B. yothersi* populations ranged from 0.36 to 1.31 mg L^−1^ and 1.22 to 4.99 mg L^−1^, respectively. RR_50_ values ranged from 0.77‐ to 2.79‐fold. The highest RR_50_ values were observed in the Barretos (2.79‐fold) and Monte Alegre de Minas (2.53‐fold) populations. The remaining populations had RR_50_ values <2.02‐fold. For seven populations—Bebedouro, Reginópolis, Tabatinga, Pirajuí‐501, Pirajuí‐502, Pirajuí‐509 and Nova Europa—the 95% confidence intervals for RR_50_ included the value 1, indicating no significant difference from the reference population (Table 2).

**Figure 3:**
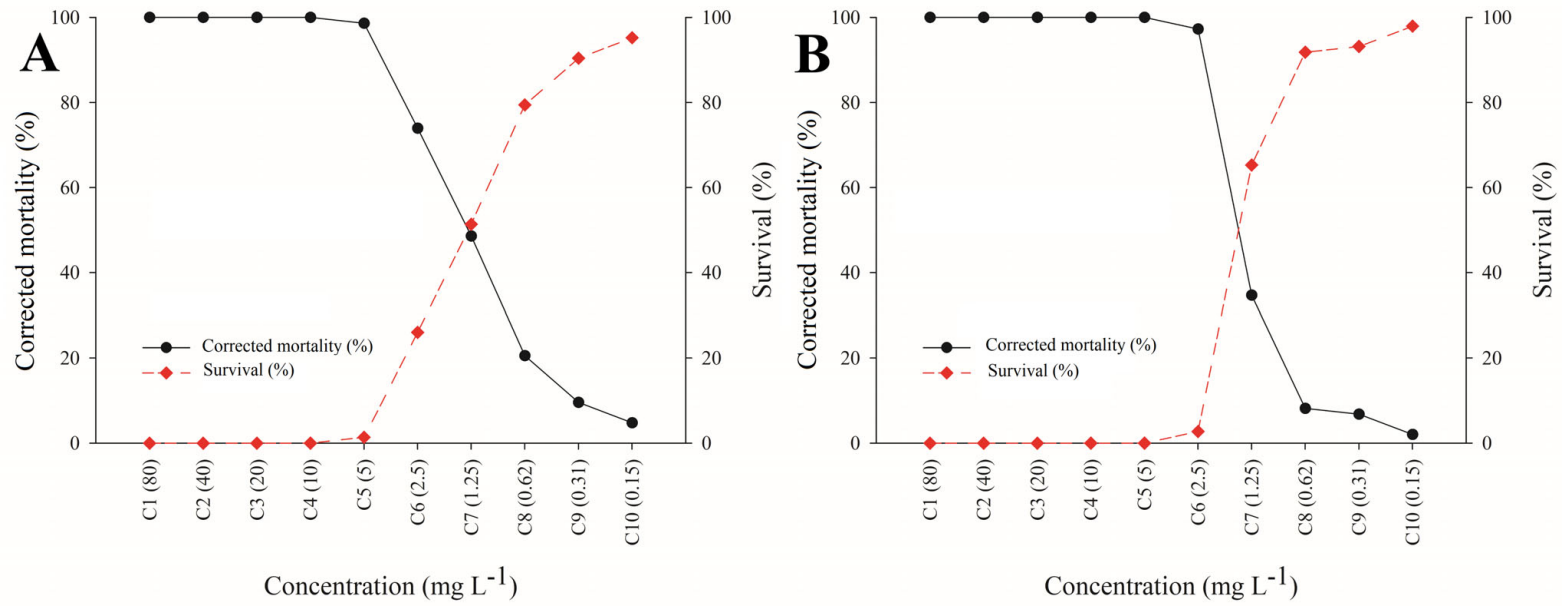
Concentration–response curves of *Brevipalpus yothersi* evaluated 48 h postexposure to cyflumetofen in the two populations with the highest resistance ratios. (A) Concentration *versus* corrected mortality and concentration *versus* survival curves (red dashed line) for the Monte Alegre de Minas population. (B) Concentration *versus* corrected mortality and concentration *versus* oviposition curves (red dashed line) for the Barretos population.

Mortality rates were recorded for each population at all tested concentrations. Overall, mortality trends were consistent with those of the reference population, as shown in Figs 2(A) and 3. Complete mortality (100%) was achieved at concentrations between 5.00 and 80.00 mg L^−1^—covering the recommended field application rate (80.00 mg L^−1^)—with the exception of the Monte Alegre de Minas population, which showed 98.63% mortality at 5.00 mg L^−1^ [Fig. 3(A)].

Oviposition also was monitored across all populations and concentrations. The results mirrored the trend observed in the reference population [Fig. 2(B)]. At concentrations between 2.50 and 80.00 mg L^−1^, oviposition was reduced by 92%–100%, except in the Monte Alegre de Minas, Itápolis and Barretos populations, for which reductions at 2.50 mg L^−1^ were 90.21%, 78.53% and 78.13%, respectively. In the reference population, larval emergence was detected after the final assessment (12 days postexposure) only at the lowest concentrations (0.15–0.62 mg L^−1^), with 40.74% to 83.95% of eggs remaining viable [Fig. 2(B)].

### 3.2 Diagnostic concentration for monitoring susceptibility of *B. yothersi* field populations

**Figure 4:**
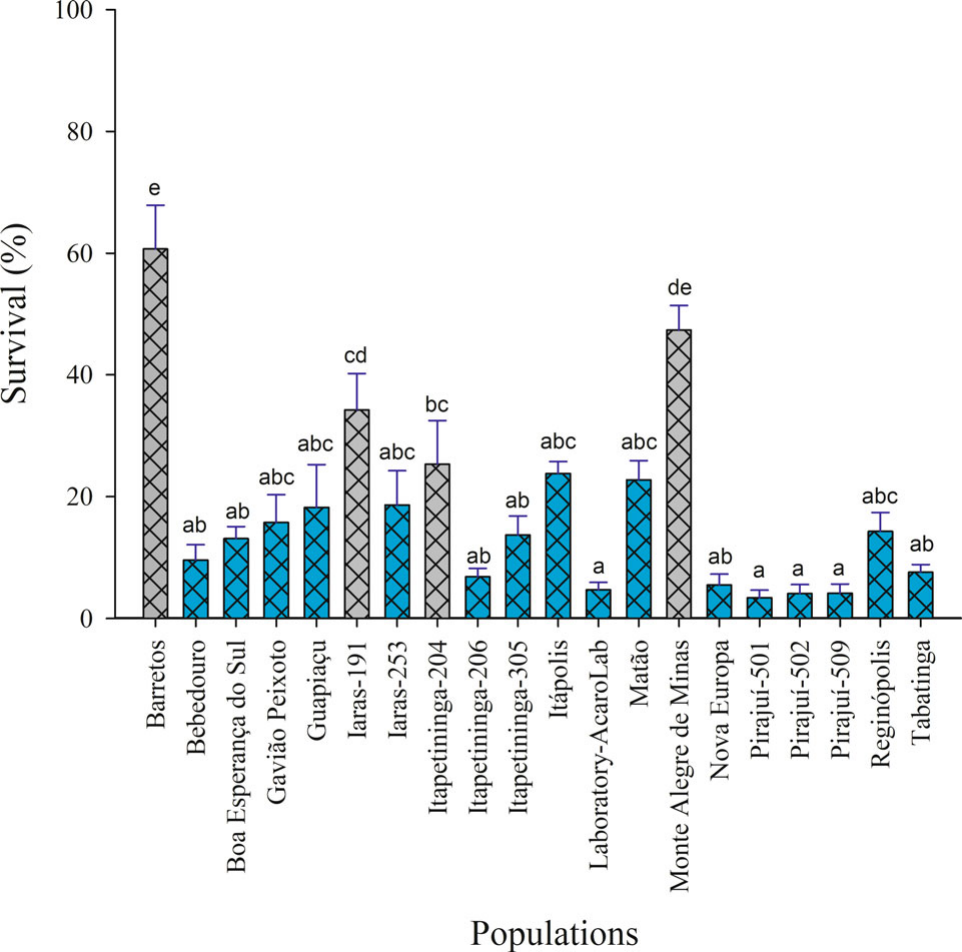
Survival percentages of *Brevipalpus yothersi* females 48 h postexposure to the diagnostic concentration of 1.63 mg L^−1^ of cyflumetofen. Bars followed by different letters indicate statistically significant differences among populations at the 95% confidence level, according to Tukey's honestly significant difference test. Five homogeneous groups were identified, within which no significant differences were observed. Bars shaded with the same color as the Laboratory–AcaroLab population represent populations that are statistically equivalent to the reference.

The diagnostic concentration established to assess *B. yothersi* susceptibility to cyflumetofen was 1.63 mg L^−1^ (95% CI = 1.37–2.04), as shown in Table 2. This concentration resulted in 95.30% (±1.24) mortality in the Laboratory‐AcaroLab reference population (Table 3). A total of 19 field populations were evaluated at this diagnostic concentration using adult females, and 78.94% of the populations were classified as susceptible to cyflumetofen at 1.63 mg L^−1^ (Table 3; Fig. 4).

**Table 3:** Survival (%) and oviposition (%) of *Brevipalpus yothersi* populations after 48‐h exposure to a diagnostic concentration of 1.63 mg L^−1^ cyflumetofen

No	Population	Survival (%) ±SE* ^,^ ^†^	95% CI (lower–upper limit)^‡^	N° eggs/control^§^	Oviposition (%) ±SE* ^,^ ^†^	95% CI (lower–upper limit)^‡^
1	Laboratory‐AcaroLab	4.70 ± 1.24 ^a^	1.52–7.88	22.67	11.77 ± 2.94 ^abc^	4.20–19.33
2	Bebedouro	9.59 ± 2.53 ^ab^	3.10–16.08	24.67	9.46 ± 2.90 ^ab^	2.01–16.91
3	Guapiaçu	18.24 ± 7.00 ^abc^	0.24–36.24	26.33	16.46 ± 5.69 ^abcd^	1.83–31.08
4	Monte Alegre de Minas	47.37 ± 4.06 ^de^	36.92–57.82	23.83	34.97 ± 7.64 ^de^	15.34–54.59
5	Itápolis	23.81 ± 1.95 ^abc^	18.80–28.82	29.50	31.64 ± 6.76 ^cde^	14.26–49.02
6	Barretos	60.70 ± 7.19 ^e^	42.21–79.19	26.67	37.50 ± 6.85 ^e^	19.90–55.10
7	Reginópolis	14.29 ± 3.12 ^abc^	6.27–22.30	23.17	10.79 ± 3.10 ^abc^	2.82–18.77
8	Itapetininga‐204	25.35 ± 7.15 ^bc^	6.96–43.73	28.17	5.92 ± 1.98 ^ab^	0.83–11.01
9	Itapetininga‐206	6.85 ± 1.37 ^ab^	3.33–10.37	24.20	6.90 ± 1.38 ^ab^	3.35–10.44
10	Itapetininga‐305	13.70 ± 3.12 ^ab^	5.67–21.73	26.17	14.01 ± 2.55 ^abc^	7.46–20.56
11	Tabatinga	7.59 ± 1.27 ^ab^	4.32–10.86	25.83	5.16 ± 1.63 ^a^	0.97–9.35
12	Boa Esperança do Sul	13.10 ± 1.97 ^ab^	8.03–18.18	16.50	10.10 ± 3.00 ^ab^	2.40–17.80
13	Iaras‐191	34.25 ± 5.97 ^cd^	18.90–49.60	25.70	26.62 ± 6.78 ^bcde^	9.19–44.05
14	Iaras‐253	18.62 ± 5.63 ^abc^	4.15–33.09	26.33	13.92 ± 3.35 ^abc^	5.31–22.53
15	Matão	22.76 ± 3.16 ^abc^	14.63–30.88	24.33	11.65 ± 1.96 ^abc^	6.60–16.69
16	Gavião Peixoto	15.76 ± 4.55 ^abc^	4.05–27.46	23.33	16.43 ± 4.62 ^abcd^	4.56–28.30
17	Pirajuí‐501	3.40 ± 1.25 ^a^	0.18–6.62	27.20	6.75 ± 2.21 ^ab^	1.06–12.43
18	Pirajuí‐502	4.08 ± 1.49 ^a^	0.25–7.91	25.33	7.24 ± 2.37 ^ab^	1.14–13.33
19	Pirajuí‐509	4.14 ± 1.51 ^a^	0.25–8.03	26.83	3.11 ± 1.15 ^a^	0.16–6.05
20	Nova Europa	5.52 ± 1.74 ^ab^	1.03–10.00	25.20	4.64 ± 1.22 ^a^	1.49–7.78

Each population was represented by 150 females in the bioassays.

* Means (%) ± SE of survival (%) and oviposition (%) are provided for each population.

^†^ Means (%) ± SE followed by the same letter are not significantly different at the 5% level by Tukey's honestly significant difference test.

^‡^ A 95% confidence interval is provided for each mean (%) (lower–upper limit).

^§^ Average number of eggs laid by 25 females over 48 h in the control treatment for each population.

However, significant differences in susceptibility were detected among populations at the 95% confidence level (Tukey's HSD test), particularly in those collected from Barretos, Iaras‐191, Itapetininga‐204 and Monte Alegre de Minas (Fig. 4). Female survival at the diagnostic concentration ranged from 3.40% to 60.70% (ANOVA: *F* = 14.59; df = 19; *P* < 0.001; Kruskal–Wallis: *H* = 79.38; df = 19; *P* < 0.001) (Table 3). The highest survival rates were observed in Barretos (60.70%) and Monte Alegre de Minas (47.37), whereas the lowest were recorded in the Pirajuí populations (ranging from 3.40% to 4.14%) (Table 3; Fig. 4).

Oviposition rates at the diagnostic concentration ranged from 3.11% to 37.50%, with the highest recorded in the Barretos population (Table 3). Significant differences were observed among populations for oviposition (ANOVA: *F* = 6.48; df = 19; *P* < 0.001; Kruskal–Wallis: *H* = 65.81; df = 19; *P* < 0.001).

**Figure 5:**
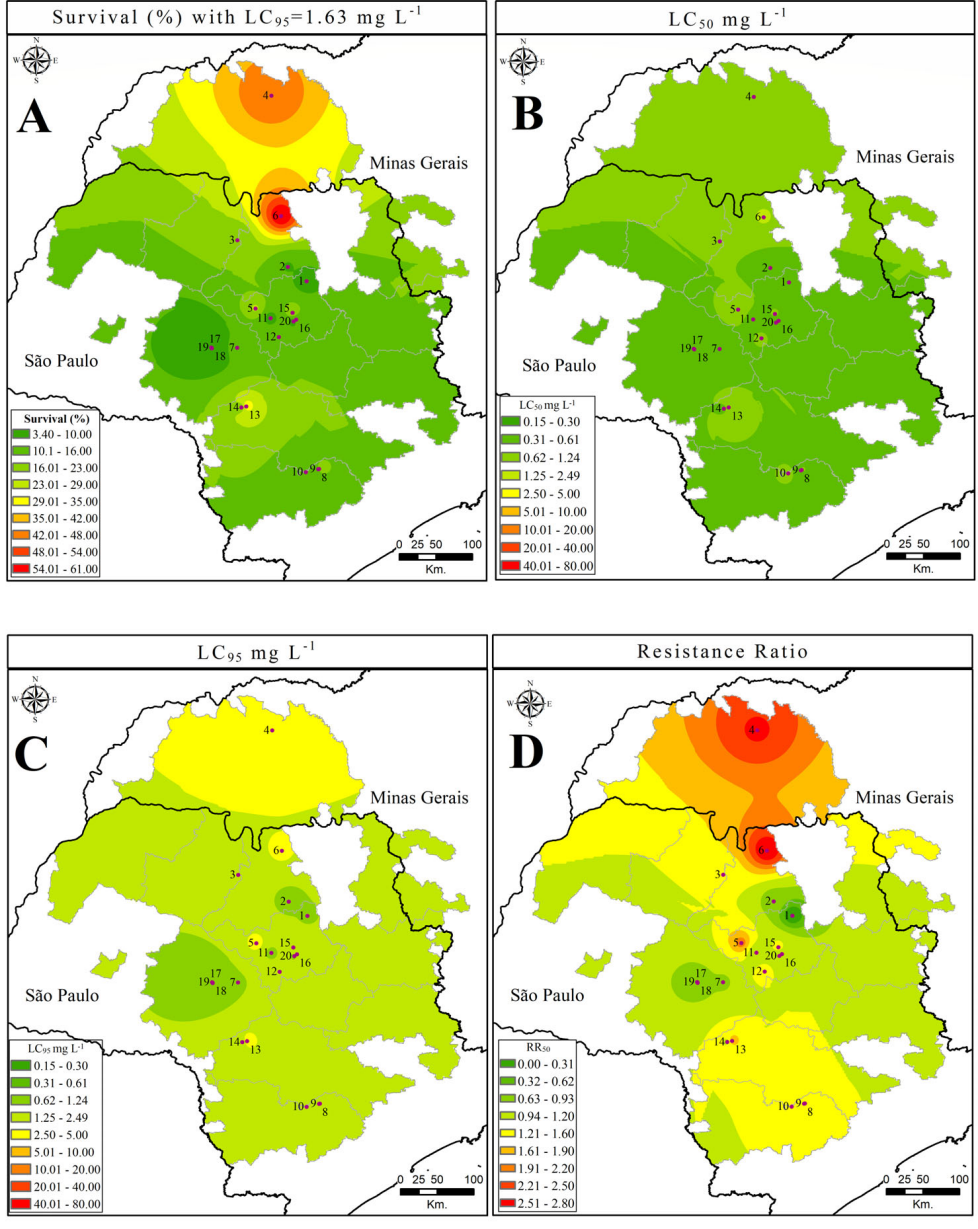
Heat map of the resistance status of *Brevipalpus yothersi* populations to cyflumetofen across the Brazilian citrus belt. (A) Survival (%) map based on exposure to the diagnostic concentration of 1.63 mg L^−1^. (B) Map displaying median lethal concentration (LC_50_) values for each population. (C) Map displaying 95% lethanlity (LC_95_) values for each population. (D) Map showing resistance ratios (RR_50_), based on LC_50_, for each population. Area colors: green, low suitability; yellow, moderate suitability; and red, high suitability for resistance development.

The use of cyflumetofen across the Brazilian citrus belt highlights the need to identify areas where resistance may first emerge. To address this, spatial analysis was conducted using arcgis software to generate thematic maps illustrating susceptibility patterns among 20 *B. yothersi* populations (including the reference), based on the following parameters: survival at the diagnostic concentration [Fig. 5(A)], LC_50_ [Fig. 5(B)], LC_95_ [Fig. 5(C)] and resistance ratio (RR_50_) [Fig. 5(D)].

In Fig. 5(A), survival rates at 1.63 mg L^−1^ ranged from 3.40% to 60.70%. The highest survival (>47.37%) occurred in the northern citrus belt, notably in Monte Alegre de Minas (Population 4) and Barretos (Population 6), indicating reduced susceptibility in these areas (Table 3). Figure 5(B) shows LC_50_ values categorized by tested concentrations. The green‐shaded regions represent populations that required <1/32 of the recommended field rate to reach 50% mortality. Figure 5(C) presents LC_95_ values, with most areas colored in green or yellow, indicating that a concentration of ≈1/16 of the field rate was sufficient to reach 95% mortality.

The RR_50_ map [Fig. 5(D)] reinforces this geographic pattern. Although all populations showed RR_50_ values <2.79, three susceptibility zones were identifiable: green for low, yellow for moderate and red for high resistance potential. The highest RR_50_ values were recorded in Monte Alegre de Minas (Population 4, RR_50_ = 2.53), with an influence area of ≈9503.34 km^2^, and in Barretos (Population 6, RR_50_ = 2.79), covering ≈1963.50 km^2^. These red‐marked areas represent potential resistance hotspots, which are likely to be linked to the more intensive use of cyflumetofen in these regions.

## 4 DISCUSSION

The introduction of cyflumetofen, a mitochondrial complex II electron transport inhibitor (METI), offers an opportunity to proactively manage resistance to both older synthetic acaricides and other METI‐type compounds used in the Brazilian citrus belt. The present results indicate that cyflumetofen is effective in controlling *Brevipalpus yothersi* populations, including those previously classified as moderately resistant to propargite at LC_95_ levels.^16^ After 48 h of exposure, LC_50_ values ranged from 0.47 mg L^−1^ for the reference population (Laboratory–AcaroLab) to 1.31 mg L^−1^ for field populations—both far below the recommended field concentration of 80 mg L^−1^.

The highest resistance ratios at LC_50_—although still low (<2.79‐fold)—were recorded in the Barretos, Monte Alegre de Minas and Itápolis populations (Table 2). These ratios are not expected to significantly compromise the efficacy of cyflumetofen in rotation schemes involving different MoAs, particularly as 100% mortality was observed in these populations at 1/8 of the recommended concentration (Fig. 3). Nonetheless, the elevated survival rates observed in Barretos and Monte Alegre de Minas at the diagnostic concentration (Table 3; Fig. 4) suggest the need for a more detailed toxicological evaluation. This is especially relevant as diagnostic concentration bioassays may underestimate actual resistance levels under field conditions.

Probit analysis revealed slope values >2.0 for all tested *B. yothersi* populations (Table 2), indicating a homogeneous susceptibility structure to cyflumetofen. This suggests a relatively uniform response among individuals within each population, which supports predictable field performance of the acaricide.^26^, ^31^ The Barretos population exhibited the highest slope (5.35), indicating a highly uniform response, whereas Monte Alegre de Minas had the lowest slope (2.65), still within the homogeneous range. These findings suggest the absence of subpopulations with differing resistance levels—an early warning signal in resistance evolution. The observed homogeneity may result from limited selective pressure from cyflumetofen in the studied areas or from its rational and strategic use. However, continuous resistance monitoring remains essential, as the introduction of migrant individuals or repeated use of the product could rapidly shift population structure and select for resistance alleles.^26^, ^32^

Oviposition is a critical parameter for evaluating acaricide efficacy, as it directly influences reproductive output and potential population growth. Even when female mortality is not absolute, reduced oviposition can significantly suppress *B. yothersi* population establishment and persistence in the field. In this context, the reduced oviposition observed after cyflumetofen exposure suggests a strong sublethal effect, contributing to population control despite partial survival.^33^, ^34^ However, it is important to recognize that incipiently resistant populations may gradually regain reproductive capacity over time, particularly under continued selection pressure. Resistance not only increases female survival, but also may restore oviposition, posing a threat to the long‐term efficacy of cyflumetofen. Therefore, monitoring oviposition over successive generations could serve as an early indicator of resistance development.^35^, ^36^

Additionally, even oviposition rates <2% at the recommended field concentration (80 mg L^−1^) may be sufficient to sustain new generations, particularly under favorable climatic conditions and in the presence of abundant host plants. Therefore, control strategies must account not only for initial mortality but also for the reproductive capacity of surviving individuals. This underscores the importance of continuous resistance monitoring and the implementation of IPM strategies, including the rotation of acaricides with distinct MoAs and the adoption of cultural practices that restricts population growth.^34^ The findings from bioassays on both lethal and sublethal effects of cyflumetofen against *B. yothersi* [Fig. 3(B)], in combination with data from previous studies on nontarget organisms, can contribute to the development of more effective IPM strategies across diverse agricultural systems.

Our results demonstrated that *B. yothersi* oviposition declined significantly with increasing concentrations of cyflumetofen. At the sublethal concentration corresponding to LC_30_ (0.31 mg L^−1^), oviposition was reduced by 35.29% [Fig. 3(B)]. Similar sublethal effects have been observed with other acaricides tested on the same populations. For instance, exposure to propargite at LC_20_ (22.5 mg L^−1^) reduced oviposition by ≤52.17%.^16^ These results align with findings from other studies involving mite pests. Ahmed and Abdelwines^37^ reported a 30.61% reduction in fecundity in *Panonychus citri* (McGregor) (Acari: Tetranychidae) after exposure to cyflumetofen at LC_20_ (2.79 mg L^−1^) in citrus orchards in Tanta, Egypt. Likewise, Mokhtari *et al*.^38^ showed that LC_30_ (1.45 mg L^−1^) of cyflumetofen applied to *Tetranychus urticae* Koch (Acari: Tetranychidae) in Iran caused an 85.94% reduction in fecundity and significant declines in key demographic parameters, including the intrinsic rate of increase (*r*_m_).

Comparable findings were reported by Saber *et al*.^39^ for another *T. urticae* population, where exposure to LC_25_ (0.45 mg L^−1^) of cyflumetofen led to an 81.37% reduction in fecundity. Havasi *et al*.^40^ found that cyflumetofen at LC_30_ had adverse effects on two phytoseiid predatory mites—*Phytoseiulus persimilis* Athias‐Henriot (LC_30_ = 4767 mg L^−1^) and *Neoseiulus californicus* (McGregor) (LC_30_ = 5921 mg L^−1^)—both collected in Tehran, Iran; fecundity was reduced by 22.74% and 11.99%, respectively. In a later study, a sublethal dose of cyflumetofen at LC_30_ (1367.9 mg L^−1^) for *Amblyseius swirskii* Athias‐Henriot (Acari: Phytoseiidae), also collected in Tehran, significantly lowered demographic parameters, including *r*_m_, and reduced fecundity by 31.30%.^41^ Likewise, in China, *P. citri* females exposed to cyflumetofen at LC_30_ (9.47 mg L^−1^) exhibited significantly lower fecundity compared to controls.^42^

Additionally, cyflumetofen at 5 mg L^−1^ was found to induce autophagy and cell death in the midgut of worker honey bees, *Apis mellifera* L. (Hymenoptera: Apidae), after just 24 h of exposure.^43^

The varying responses of *B. yothersi* populations to cyflumetofen may be attributed to differences in historical selection pressure across orchards. Previous studies have shown that resistance levels often vary among populations as a result of differing selection intensities.^44^, ^45^ This suggests that populations from Barretos, Monte Alegre de Minas and Itápolis may have been subjected to stronger selection pressure from cyflumetofen use compared to others in this study. Without the implementation of proactive resistance management strategies, the likelihood of resistance development and control failures will increase. Therefore, resistance management should be guided by localized monitoring data, as biogeographical context also influences variability in susceptibility (Table 1; Fig. 5).

Spatial analysis of *B. yothersi* susceptibility to cyflumetofen revealed clear geographic variation in responses across the Brazilian citrus belt (Fig. 5). Although most populations displayed low resistance ratios (RR_50_ < 2.79‐fold), indicating overall susceptibility, potential resistance hotspots were identified, particularly in the northern region [Fig. 5(D)]. Populations from Barretos (Population 6) and Monte Alegre de Minas (Population 4) exhibited the highest RR_50_ values and the largest influence areas, which is likely to reflect more frequent use of cyflumetofen in these locations.^9^, ^32^

Zones with moderate resistance potential (yellow areas) also warrant attention, as they may represent transitional zones under increasing selection pressure. Resistance management practices—including rotation with acaricides of differing MoAs and integration with biological control measures—should be adopted in these areas to delay resistance evolution.^9^, ^32^, ^34^

The use of ArcGIS for spatial analysis proved to be a flexible and effective tool for resistance mapping in citrus orchards, as it has in other agricultural contexts.^30^ Fig. 5 offers a valuable resource for guiding the strategic use of cyflumetofen in *B. yothersi* management. These findings also support future monitoring efforts by identifying priority areas for susceptibility tracking and early intervention. In regions where field collections were not performed but that fall within or near zones of moderate‐to‐high resistance potential, a preventive approach is recommended. This includes routine assessment of cyflumetofen efficacy and restrictions on its continuous application. While the arcgis model allows for spatial extrapolation of susceptibility trends, further sampling will be necessary to validate predictions and refine regional resistance management strategies.^30^

Significant differences in susceptibility to cyflumetofen were observed among the 19 *B. yothersi* populations collected from 14 citrus‐producing locations. All populations exhibited low resistance levels, confirming that cyflumetofen remains an effective option for controlling *B. yothersi* in citrus orchards. To preserve its long‐term efficacy, cyflumetofen should be used as part of an acaricide rotation strategy involving compounds with distinct MoAs. Moreover, spray programs should be tailored according to regional resistance profiles to ensure the continued success of control efforts in Brazilian citrus production systems.

## FUNDING INFORMATION

This research is part of the PhD thesis of Hector Alonso Escobar‐Garcia, funded by the Coordination for the Improvement of Higher Education Personnel – Brazil (CAPES) – Financing Code 001, and conducted at the AcaroLab laboratory, São Paulo State University (UNESP), Jaboticabal Campus, Brazil.

## CONFLICT OF INTEREST

The authors declare no conflict of interest.

## Data Availability

The data that support the findings of this study are available from the corresponding author upon reasonable request.
